# Rumination in Man

**Published:** 1887-05

**Authors:** 


### Rumination in Man.

The patient, a child of eleven, had contracted the habit of causing food to return into the mouth, in order to repeat the taste of any aliments he preferred. In order to effect this, he first recalled the taste of whatever aliment he had most enjoyed in his meal, and by contracting his abdominal muscles caused the particular aliment of which he wished to repeat the taste to return into the mouth. He occasionally repeated this action a second time. On examination, the stomach was found to be slightly dilated, but made no rumbling sound; the child was pale and rather anæmic. His parents had tried every means of curing this habit. When a young child he lived in the country, and milk was his principle article of diet. The stomach became overloaded, and regurgitations ensued; the child became accustomed to these regurgitations, which finally caused a pleasurable sensation, and the habit of merycism was thus induced. M. le Juge de Sevrais proposes to submit the patient to a course of hydrotherapy, to wash the stomach out with Faucher's apparatus, and to apply actual cautery to the epigastric region. If this case of merycism be a neurosis, age and reason should cure such an affection.

